# Differences in EEG complexity of cognitive activities among subtypes of schizophrenia

**DOI:** 10.3389/fpsyt.2025.1473693

**Published:** 2025-02-05

**Authors:** Hang Qi, Jilin Zou, Zhenzhen Yao, Gaofeng Zhao, Jing Zhang, Chunlei Liu, Min Chen

**Affiliations:** ^1^ School of Psychology, Qufu Normal University, Qufu, China; ^2^ Department of Psychology, School of Education, Linyi University, Linyi, Shandong, China; ^3^ Clinical Psychology Department, Shandong Mental Health Center, Jinan, China; ^4^ Geriatrics Department, Shandong Daizhuang Hospital, Jining, China; ^5^ School of Mental Health, Jining Medical University, Jining, China

**Keywords:** schizophrenia, working memory, EEG complexity, frequency bands, sample entropy

## Abstract

**Introduction:**

The neural mechanisms that underpin cognitive impairments in patients with schizophrenia remain unclear. Previous studies have typically treated patients as a homogeneous group, despite the existence of distinct symptom presentations between deficit and non-deficit subtypes. This approach has been found to be inadequate, necessitating separate investigation.

**Methods:**

This study was conducted at Daizhuang Hospital in Jining City, China, from January 2022 to October 2023. The study sample comprised 30 healthy controls, 19 patients with deficit schizophrenia, and 19 patients with non-deficit schizophrenia, all aged between 18 and 45 years. Cognitive abilities were evaluated using a change detection task. The NeuroScan EEG/ERP System, comprising 64 channels and utilising standard 10-20 electrode placements, was employed to record EEG signals. The multiscale entropy and sample entropy of the EEG signals were calculated.

**Results:**

The healthy controls demonstrated superior task performance compared to both the non-deficit (p < 0.001) and deficit groups(p < 0.001). Significant differences in multiscale entropy between the three groups were observed at multiple electrode sites. In the task state, there are significant differences in the sample entropy of the β frequency band among the three groups of subjects. Under simple conditions of difficulty, the performance of the healthy controls exhibited a positive correlation with alpha band sample entropy(r = 0.372) and a negative correlation with beta band sample entropy (r = -0.411). Deficit patients demonstrated positive correlations with alpha band sample entropy (r = 0.370), whereas non-deficit patients exhibited negative correlations with both alpha and beta band sample entropy (r = -0.451, r = -0.362). Under difficult conditions of difficulty, the performance of healthy controls demonstrated a positive correlation with beta band sample entropy (r = 0.486). Deficit patients exhibited a positive correlation with alpha band sample entropy (r = 0.351), while non-deficit patients demonstrated a negative correlation with beta band sample entropy (r = -0.331).

**Conclusion:**

The results of this study indicate that cognitive impairment in specific subtypes of schizophrenia may have distinct physiological underpinnings, underscoring the need for further investigation.

## Introduction

Schizophrenia is a significant psychiatric disorder that is characterised by substantial functional impairments and cognitive deficits. However, the underlying pathophysiology of this disorder remains unclear (1–3). The advent of new technologies has enabled researchers to identify an increasing number of neuroimaging differences between patients with schizophrenia and healthy individuals. However, substantial variability exists in cognitive abilities and symptom presentations among different subtypes of schizophrenia, which can lead to conflicting research conclusions and complicate the interpretation of results (4–6). Consequently, a comparison of the cognitive abilities and brain activities between different subtypes of schizophrenia and healthy populations facilitates a more accurate distinction between symptomatology and an enhanced comprehension of the physiological mechanisms underlying cognitive impairments.

Schizophrenia is characterised by the presence of distinct subtypes, which are commonly categorised internationally as deficit and non-deficit types (7). The former primarily exhibits negative symptoms, such as emotional blunting and social withdrawal (8, 9), while the latter is dominated by positive symptoms, including hallucinations and delusions (10–12). These subtypes not only differ in symptomatology but also in the severity of cognitive impairment (13). However, previous research has often treated schizophrenia patients as a homogeneous group, thereby overlooking the existence of significant internal subtypes, which may impede the progress of research in this field (14, 15). Moreover, neuroimaging studies that have focused on both early and chronic schizophrenia have identified potential neural markers of cognitive impairment. However, the findings indicate significant individual variations in these markers. For example, some patients display increased activity in the frontal and temporal lobes, while others exhibit decreased activity in the frontal and marginal systems (16). In the context of cognitive tasks, some patients may exhibit either enhanced or diminished P300 wave amplitudes (17). In light of the considerable individual variability in brain regions and neural signals observed among schizophrenia patients, researchers are now directing their attention towards patterns of activation and connectivity across the entire brain, rather than focusing on specific regional activities alone (15).

The human brain is acknowledged as a sophisticated network of interlinked regions that continuously process and integrate temporally synchronised information during cognitive processes (18, 19). Consequently, the temporal fluctuations exhibited by brain electrical signals reflect nonlinear dynamic changes. The assessment of brain signal complexity offers a novel approach to elucidating the intrinsic neural network mechanisms that underpin a range of neurophysiological processes (20).

At present, the most frequently employed methodology for assessing the complexity of brain signals is Multiscale Entropy (MSE), as proposed by Costa et al. This approach entails the coarse-graining of signals and the computation of Sample Entropy (SampEn) on novel time series, thereby effectively conveying complexity profiles across disparate time scales (21–24). However, recent studies put forth alternative perspectives that challenge the effectiveness of MSE in capturing signal complexity across different time spans and ranges (25). Therefore, the objective of this study is to compute Sample Entropy separately for signals in different frequency bands, with the aim of gleaning dynamic variation information across spatiotemporal domains. The processing of information in the brain is dependent on the dynamic interactions between neural ensembles and rhythmic activities (26). Furthermore, different frequency bands often synchronise with neural activities within various ranges (27). This synchronisation is associated with a number of cognitive functions, including attention, consciousness, working memory and perceptual grouping. Impairments to these functions have been observed in schizophrenia patients (28, 29). Schizophrenia has been demonstrated to manifest as distributed disturbances across numerous brain regions and their interconnections, rather than as localised defects (30–34). The analysis of complexity across different frequency bands facilitates an understanding of the differences in connectivity at various levels. Prior studies have concentrated on the analysis of the complexity of raw EEG signals, with little attention paid to the complexity of signals in different frequency bands (35). The objective of this study is to address this gap in the existing literature.

This study employs two measures of EEG complexity, namely multiscale entropy (MSE) and sample entropy (SampEn), to investigate the complexity of brain function in healthy individuals and in those with different subtypes of schizophrenia. In addition, the study examines the potential correlations between these measures of complexity and cognitive performance. The following hypotheses are proposed: differences in multiscale entropy between healthy controls, deficit subtype, and non-deficit subtype during rest and task states; differences in sample entropy in the α/β frequency bands across the three groups, reflecting variations in different brain regions; and distinct correlations between task performance and sample entropy in the α/β frequency bands across the three groups.

## Methods

### Participants

A total of 19 patients with deficit-type schizophrenia and 19 patients with non-deficit-type schizophrenia, admitted to Daizhuang Hospital in Shandong Province between January 2022 and October 2023, were selected as the study group(The demographic data is shown in **Table 1**). All patients included in the study met the diagnostic criteria for schizophrenia as outlined in the International Classification of Diseases, Tenth Revision (ICD-10), as confirmed by at least two senior-level psychiatrists. The patients were subsequently classified as either deficit or non-deficit schizophrenia in accordance with the Chinese version of the Schedule for the Deficit Syndrome (SDS) (36). Furthermore, 30 healthy volunteers from the surrounding communities of Daizhuang Hospital during the same period were selected as the control group. The participants were fully apprised of the nature of the study and provided informed consent, either personally or through their legal guardians. Subject selection criteria (1): According to the diagnostic criteria of ICD-10 schizophrenia, SDS is used for the classification of defective/non defective schizophrenia (2) Age range: 18-45 years old, with the first onset occurring after the age of 18; (3) Not taking medication or taking low-dose atypical antipsychotic drugs (less than 300mg chlorpromazine equivalent) for treatment, and able to cooperate in completing the examination; (4) Normal vision or corrected vision; (5) Primary school education or above; (6) Han Chinese, right-handed. Exclusion criteria: (1) History of traumatic brain injury, neurological disorders, or other major physical illnesses; (2) Convulsive electroconvulsive therapy patients; (3) History of alcohol or drug abuse or dependence; (4) Individuals with secondary psychotic symptoms caused by other organic factors or drug use; (5) Intellectual disability.

**Table 1:** Patient demographic data.

	NS group	NDS group	Health group
age	30.96 ± 6.69	31.10 ± 8.12	30.71 ± 5.31
Education age	12.14 ± 3.43	12.10 ± 3.10	12.45 ± 3.12
Sex ratio (male/female)	15/4	10/9	16/14

### Clinical assessments

The Positive and Negative Syndrome Scale (PANSS) is a widely utilised clinical scale for evaluating the symptoms of patients with schizophrenia (37). The scale comprises four sections: the General Psychopathology Scale (GPS), the Positive Scale (POS), the Negative Scale (NEG), and three supplementary scales, for a total of 33 items. Each item is scored on a 7-point scale, ranging from 1 (absence of symptoms) to 7 (extremely severe), with higher scores indicating a greater severity of symptoms. The experimental paradigm chosen was a modification of the change detection task, in which subjects were presented with stimulus material that appeared on both sides of fixation as blue or red colour blocks in several orientations (0°, 45°, 90°, 135°), with the block orientations presented randomly. On each trial, subjects were presented with a brief array of colour blocks with different orientations on each side and were asked to remember only the orientation of the left or right colour block.1 s later the blocks were tested with a test array that was either the same as or different from the original memory item. Subjects reported whether the red or blue blocks were the same in both arrays by pressing one of two buttons. The task consisted of two conditions: one colour block (1T),four colour blocks (4T). In the formal experiment, each condition was presented 80 times.

**Figure 1:**
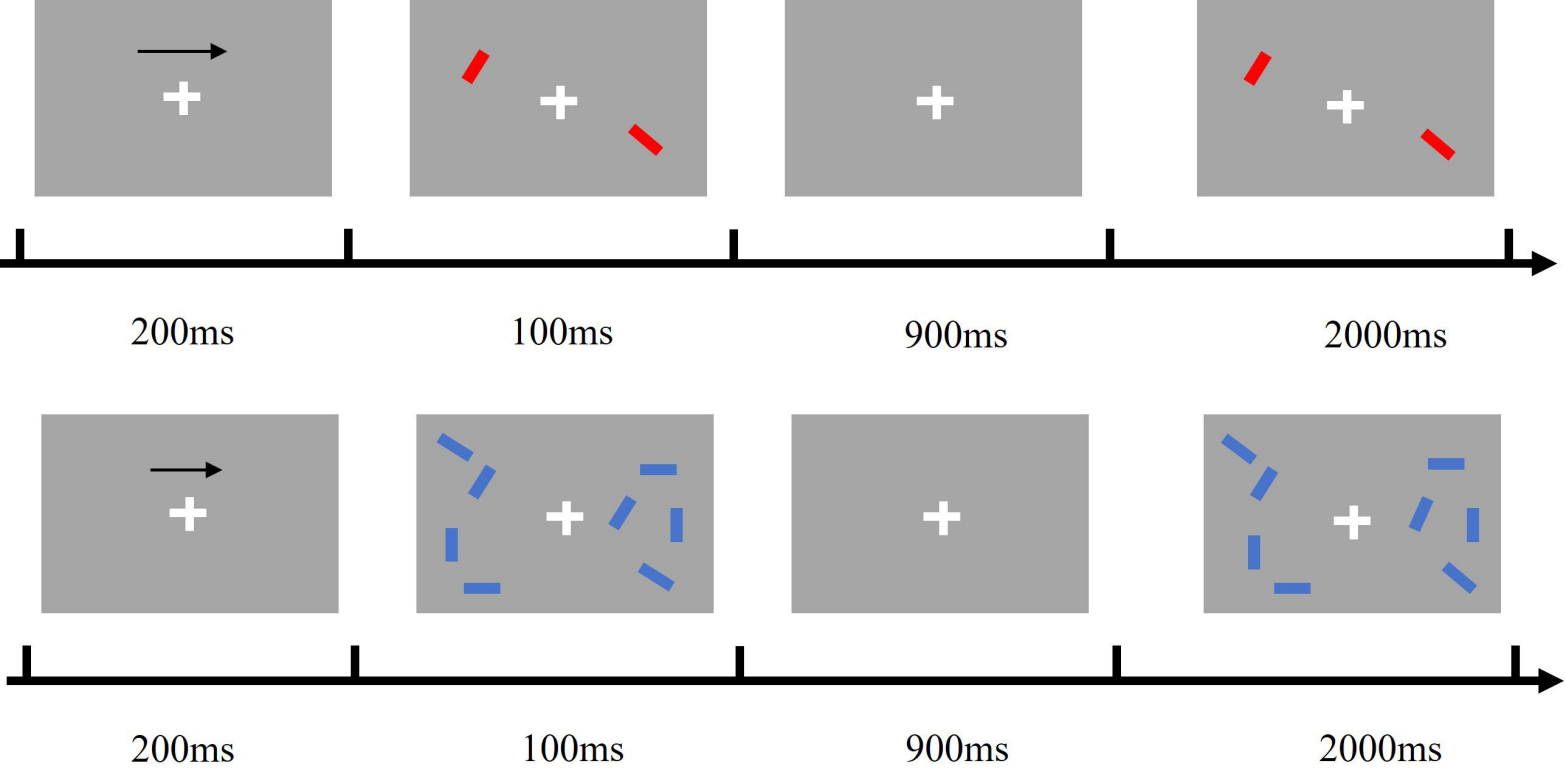
The process of change detection task.

As shown in **Figure 1**, each trial began with an arrow (200 ms, with a 50% probability that the target was on the left rather than the right side of the gaze), followed by a memory array (100 ms), a delay (blank screen, 900 ms), and a test array (2000 ms, with a 50% probability that the stimulus was the same as, rather than different from, the memory array). Subjects were asked to remember the orientation of the colour blocks in the memory array at the half-field of view (left or right) indicated by the arrow and to press one of the two response buttons during the presentation of the test array to indicate whether the orientation of the red or blue colour block blocks in the test array matched the orientation of the colour blocks in the memory array.

### EEG collection and data processing

The EEG data were collected using the NeuroScan EEG/ERP system, comprising 64 channels. A total of 64 recording electrodes were positioned in accordance with the international 10-20 system. During the recording process, the right mastoid (M2) was employed as the reference electrode, while the ground electrode was situated at FPZ on the midline of the forehead. The vertical eye movement (VEOG) was recorded at a distance of 2 cm above and below the left eye, while the horizontal eye movement (HEOG) was recorded at a distance of approximately 1 cm lateral to each eye. The sampling frequency was set to 1000 Hz, with a bandpass filter of 0.01 to 100 Hz. The subsequent offline analysis of the continuous EEG data was conducted using Matlab (R2021b) and EEGLAB (v2022). The data were re-referenced to the average reference of all electrodes, filtered between 0.1 Hz and 40 Hz, The resampling frequency is 500Hz, Artifacts such as eye movements and EMG were then removed, using Independent Component Analysis (ICA). Artifacts such as instantaneous fluctuations of more than ±50 µV between sampling points; peak-to-peak differences of more than 200 µV between 200 ms; differences between maximum and minimum amplitudes of more than ±100 µV; and fluctuations of less than 0.5 µV in 100 ms intervals were excluded. Subjects with more than 70% of remaining trials were included in the statistics.

For the EEG signals in the resting state with eyes closed, a time window of (10,000ms-180,000ms) was chosen as the computational object, a time window of 10,000 was selected for segmentation, and 500ms before each epoch was chosen as the baseline correction. Rejection of bad segments after segmentation. For the change detection task, a time window of 500 ms before the first stimulus presentation to 3,000 ms after the first stimulus presentation (-200-3,000 ms) was selected for segmentation, and 200 ms before stimulus presentation was used as the baseline correction.

### Calculation of EEG complexity

The calculation of EEG complexity employs two methods: Sample Entropy (SampEn) and Multiscale Entropy (MSE). For a given time series {x(i)} of length N, a set of vectors X(i) is constructed, with X(i) = {x(i), x(i + 1),…, x(i + m - 1)} where i = 1, 2,…, N - m + 1 and 𝑚 represents the length of the patterns extracted from the time series signals. For each X(i), the distances to other vectors are calculated as follows: d(X(i),X(j)) = max(|x(i+k)−x(j+k)|), where 0≤k≤m−1. 𝑟 denotes the allowable error, which indicates the threshold for voltage signals, and vectors X(i) and X(j) are deemed similar if the distance between them, denoted as d(X(i),X(j)), is less than or equal to r. The number of vectors with a distance less than r from X(i) is represented as A(m,r). The conditional probabilities C(m, r) and C(m + 1, r) are calculated as follows:


$$C\left(m,r\right)=\frac{\text{A}\left(m,r\right)}{\text{N}-m}$$

Sample Entropy is computed using the following formula:


$$\text{SampEn}(\text{m},\text{r})=-1\text{n}\frac{C(m,r)}{C(m+1,r)}$$

Multiscale Entropy (MSE) is the process of computing Sample Entropy after the time series has been coarse-grained according to time scales τ = 1, 2, 3,…, 20. This step is typically implemented using mean smoothing. The calculation of Sample Entropy is performed for each time scale, with the resulting values subsequently aggregated in order to obtain Multiscale Entropy.

### Statistical analysis

The data were statistically processed using the SPSS 25.0 software package. The Levene’s test was employed to assess the assumption of homogeneity of variance. For data satisfying the assumption of homogeneity, an analysis of variance was conducted, followed by the Bonferroni *post-hoc* test for paired comparisons; an independent sample t-test was used to compare between two groups. Pearson's linear correlation analysis was utilised to investigate the correlation between EEG complexity and working memory capacity. A p-value < 0.05 was considered statistically significant, indicating a difference.

## Result

### Behavioral data analysis

**Figure 2:**
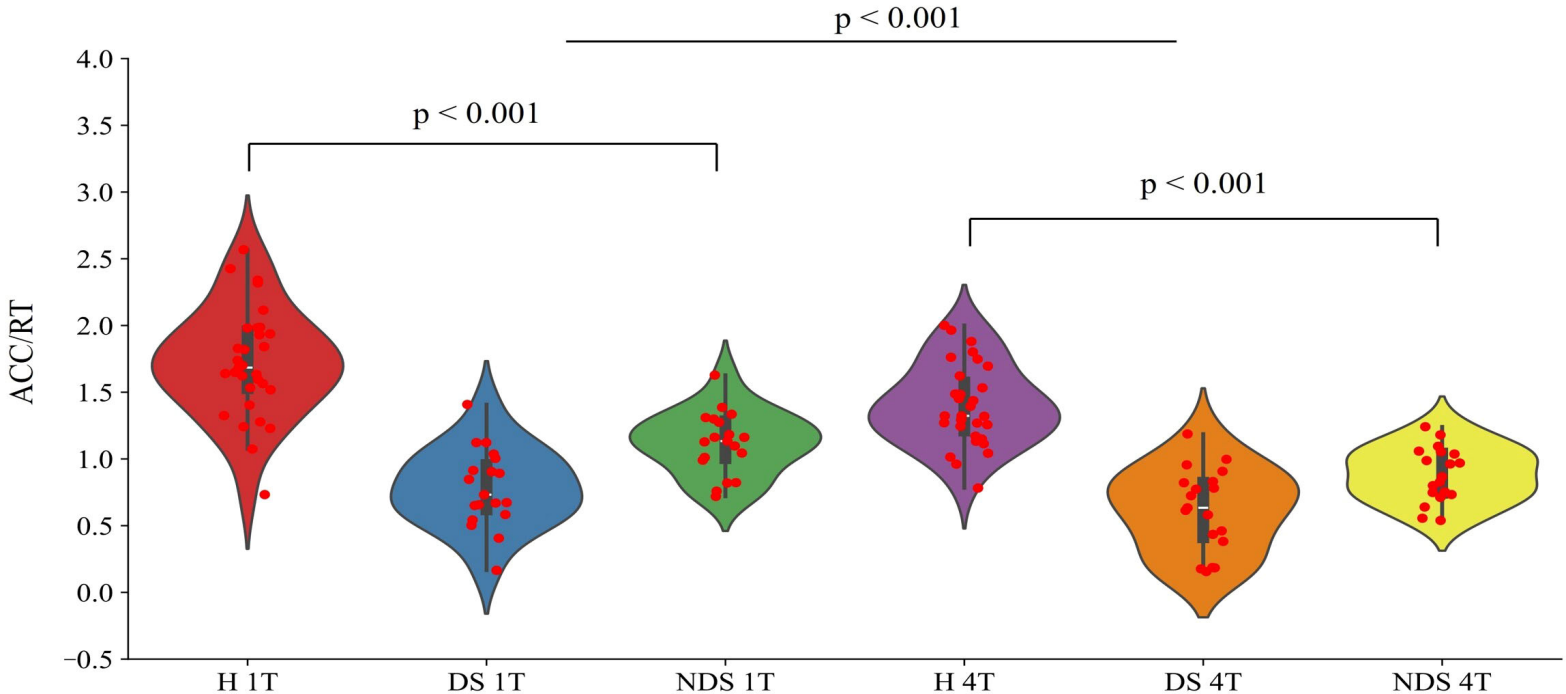
Performance ratios (ACC/RT) of the healthy group (H), deficit group (DS), and non-deficit group (NDS) in the change detection task with two task difficulties (1T/4T).

Using task difficulty (1T/4T) and group (healthy/impaired/non-impaired) as independent variables, and task performance (ACC/RT) as the dependent variable, a repeated measures two-way ANOVA was conducted on the data, with the results shown in **Figure 2**. Significant differences were observed among different groups (F(2,130) = 99.56, p < 0.001, η2p = 0.649). A Bonferroni pairwise test was performed on the groups, revealing that the healthy group's task performance was significantly higher than both the impaired group (t(96) = 11.35, p < 0.001, Cohen's d = 1.664) and the non-impaired group (t(96) = 9.21, p < 0.001, Cohen's d = 1.350). The non-impaired group significantly outperformed the impaired group (t(74) = 4.686, p < 0.001, Cohen's d = 0.760). Significant differences were also observed in task performance across varying levels of difficulty (F(1,130) = 18.288, p < 0.001, η2p = 0.145).

### Comparison of resting-state and task-state multiscale entropy

**Figure 3:**
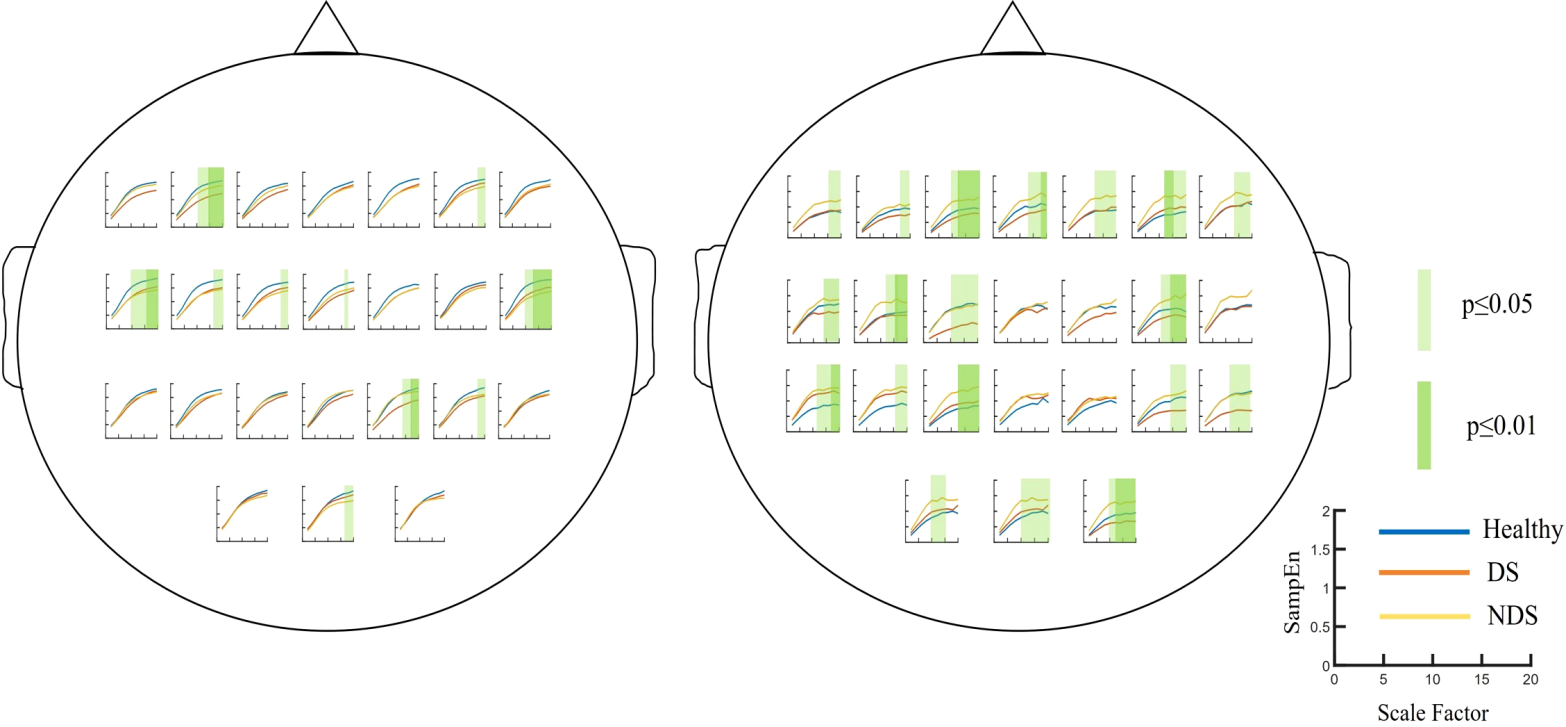
Multiscale entropy of the Healthy Group / Deficit Group / Non-Deficit Group in Resting State (Left) and Task State (Right), with significant differences highlighted in green squares.

**Figure 4:**
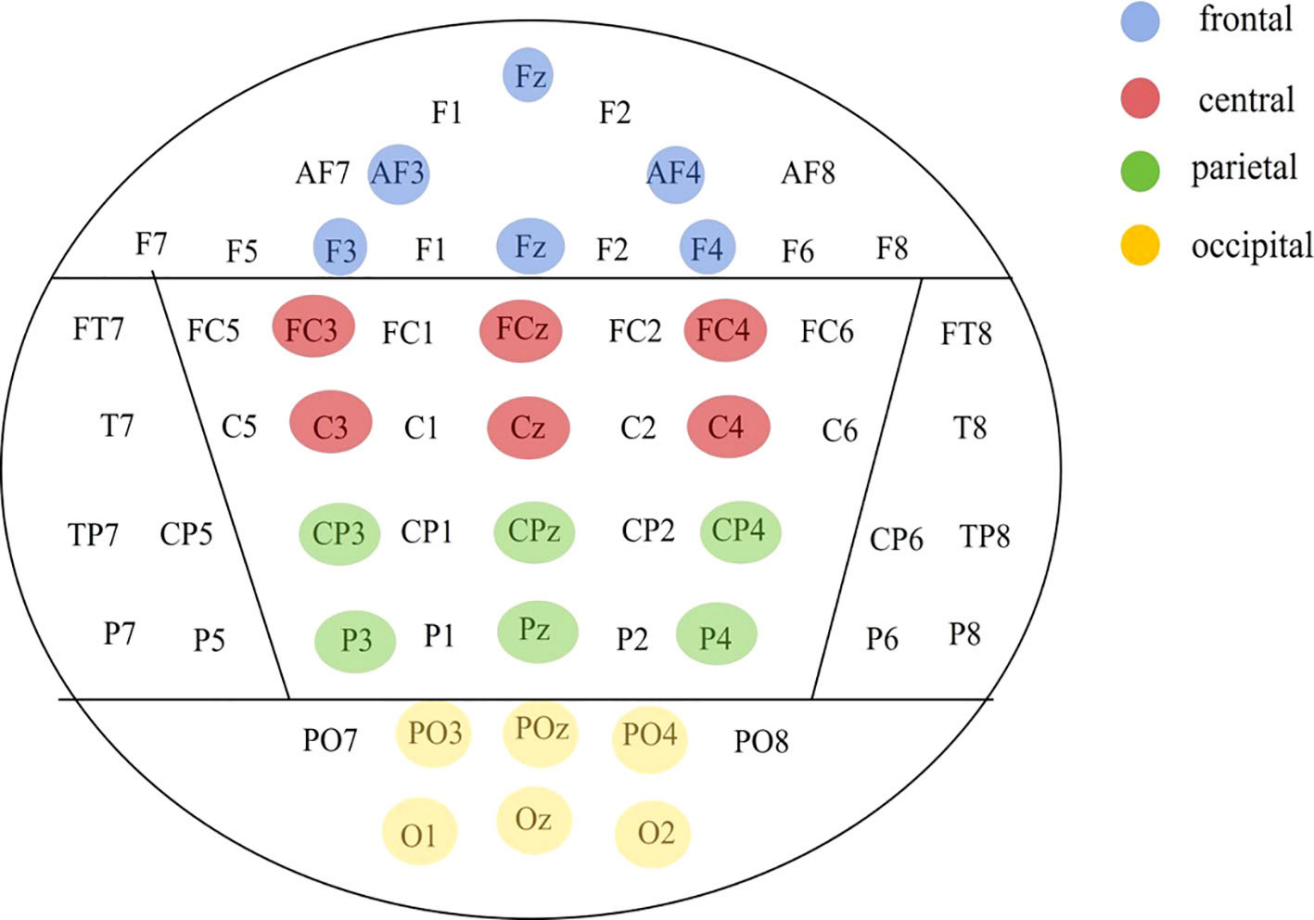
Division of brain regions based on electrode points.

The group was employed as the independent variable in a one-way ANOVA on multiscale entropy at 10-20 time scales for each electrode point during both the eyes-closed resting state and the task state. The results are displayed in **Figure 3**. Significant differences were observed in the resting state at the following electrodes: F3, C5, C6, F2, and Oz. In the task state, significant differences were observed at all electrode points with the exception of Cz, C2, Pz, and P2. The Bonferroni pairwise comparisons between the healthy and deficit groups according to the brain region (**Figure 4**) indicated that during the task state, the deficit group exhibited lower MSE in the central region compared to the healthy group (*t*(47) = -2.5, *p* = 0.016, Cohen's d = 0.518). The non-deficit group exhibited higher MSE in the frontal (t(47) = 2.99, p = 0.004, Cohen's d = 0.620), parietal (*t*(47) = 3.01, *p* = 0.004, Cohen's d = 0.624), central (*t*(47) = 3.14, *p* = 0.003, Cohen's d = 0.678), and occipital (*t*(47) = 3.27, *p* = 0.002, Cohen's d = 0.678) regions compared to the healthy group. No significant differences were identified during the resting state (**Table 2**).

**Table 2:** Independent sample t-tests of multiscale entropy(10-20) for healthy group, deficit group, and non-deficit group in resting-state and task-state conditions.

	*t_(close)_*	*P_(close)_*	*t_(task)_*	*P_(task)_*
DS				
Frontal	-0.54	0.584	-1.85	0.07
Central	-0.69	0.492	**-2.50**	**0.016**
Parietal	-0.18	0.858	0.10	0.922
Occipital	-1.28	0.206	0.22	0.825
NDS				
Frontal	-0.12	0.950	**2.99**	**0.004**
Central	-0.31	0.758	**3.14**	**0.003**
Parietal	-0.14	0.889	**3.01**	**0.004**
Occipital	-0.22	0.826	**3.27**	**0.002**

The red values indicate significant differences.

### Resting-state and task-state sample entropy in different frequency bands

**Figure 5:**
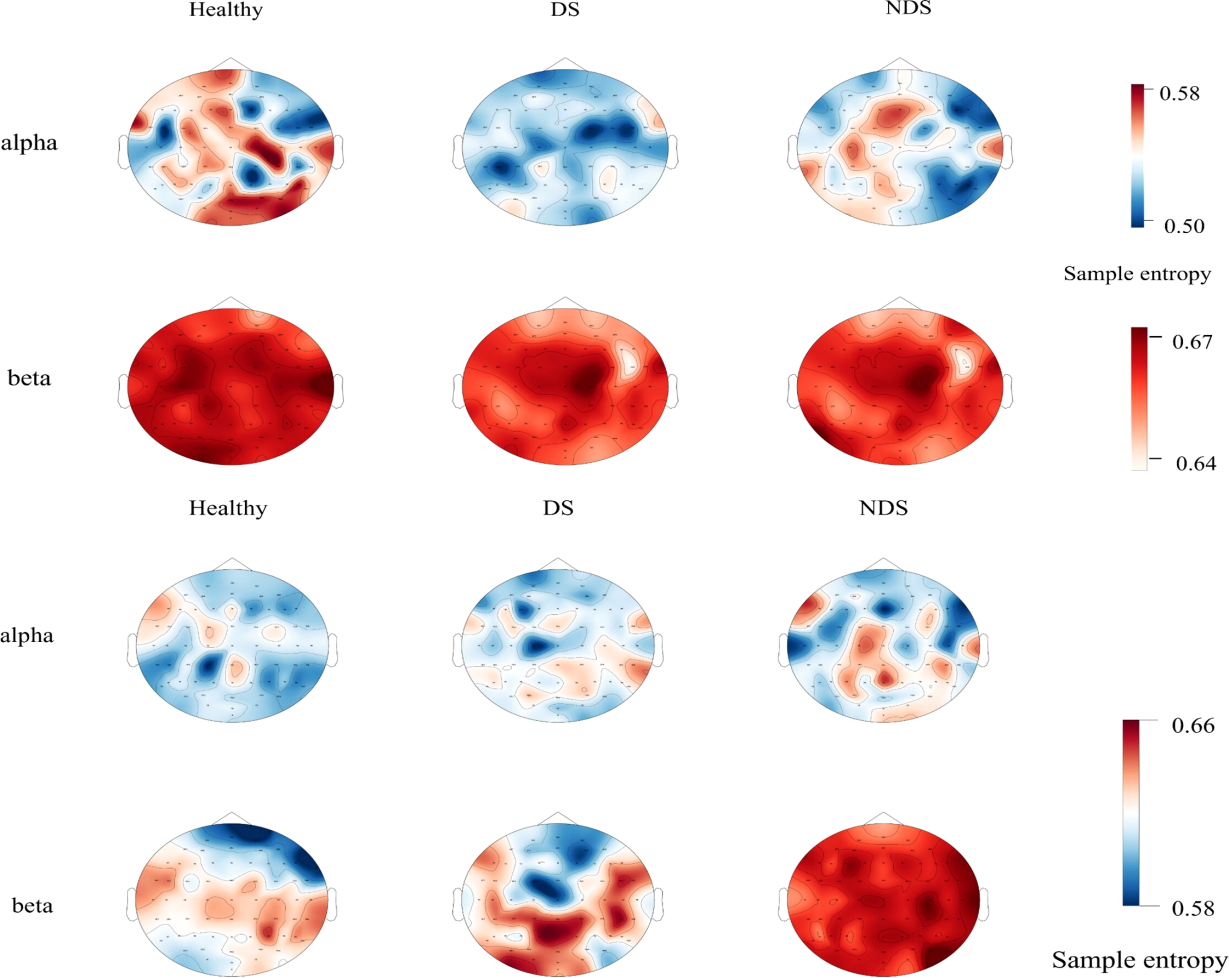
Topographic maps of sample entropy of α/β band electroencephalography in resting state (top) and task state (bottom) for the healthy group, deficit group, and non-deficit group.

**Figure 6:**
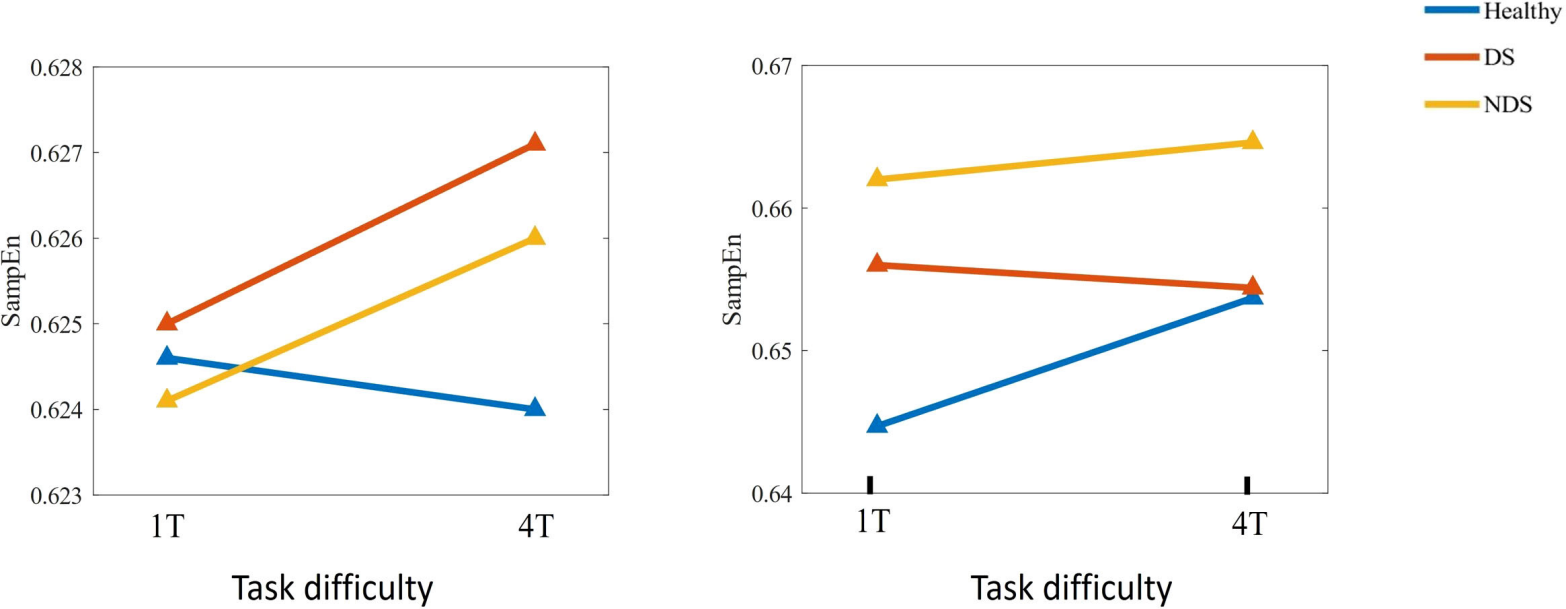
Changes in sample entropy of α (left) and β (right) frequency bands across different task difficulties for the Healthy Group / Deficit Group / Non-Deficit Group.

Further analysis was conducted on sample entropy in different frequency bands during both resting state and task state, as illustrated in **Figure 5**, **Table 3**. No significant intergroup differences were observed in the β band during the resting state. Significant differences in α band sample entropy were observed in the occipital region (*F*(2,65) = 15.28, *p* < 0.001, ηp^2^ = 0.320). The results of the Bonferroni pairwise comparisons indicated that the healthy group exhibited significantly higher α band sample entropy than both the deficit group (*t*(47) = 2.97, *p* = 0.002, Cohen's d = 0.616) and the non-deficit group (*t*(47) = 2.77, *p* = 0.004, Cohen's d = 0.574). In the β band, during the task state, the deficit group exhibited significantly higher sample entropy in the parietal region compared to the healthy group (t(47) = 2.68, p = 0.006, Cohen's d = 0.556), while the non-deficit group demonstrated higher sample entropy in the frontal (*t*(47) = 4.01, *p* < 0.001, Cohen's d = 0.831),central (*t*(47) = 2.76, *p* = 0.004, Cohen's d = 0.572), parietal (*t*(47) = 2.36, *p* = 0.018, Cohen's d = 0.489), and occipital (*t*(47) = 3.35, *p* = 0.002, Cohen's d = 0.695) regions compared to the healthy group (**Table 2**). A two-way repeated measures ANOVA on α/β band sample entropy with task difficulty and group as independent variables revealed no significant differences in the α band (**Figure 6**). In the β band, a significant main effect of group was observed (*F*(2,130) = 13.30, *p* < 0.001, ηp² = 0.198). The results of the Bonferroni pairwise comparisons revealed that the non-deficit group exhibited significantly higher β band sample entropy compared to both the healthy group (*t*(96) = 3.56, *p* < 0.001, Cohen's d = 0.522) and the deficit group (*t*(96) = 5.99, *p* < 0.001, Cohen's d = 0.522).

**Table 3:** Independent sample t-test for the entropy of EEG alpha/beta frequency bands in the task state deficit group, non-deficit group, and healthy group subjects.

	*t_(α)_*	*P_(α)_*	*t_(β)_*	*P_(β)_*
DS				
Frontal	0.24	0.812	1.02	0.314
Central	-0.50	0.610	-0.87	0.394
Parietal	1.50	0.153	**2.68**	**0.006**
Occipital	2.01	0.052	0.51	0.614
NDS				
Frontal	0.97	0.527	**4.01**	**<0.001**
Central	1.90	0.062	**2.36**	**0.018**
Parietal	0.15	0.882	**2.76**	**0.004**
Occipital	0.77	0.446	**3.35**	**0.002**

The red values indicate significant differences.

### Correlation between task performance and sample entropy

**Figure 7:**
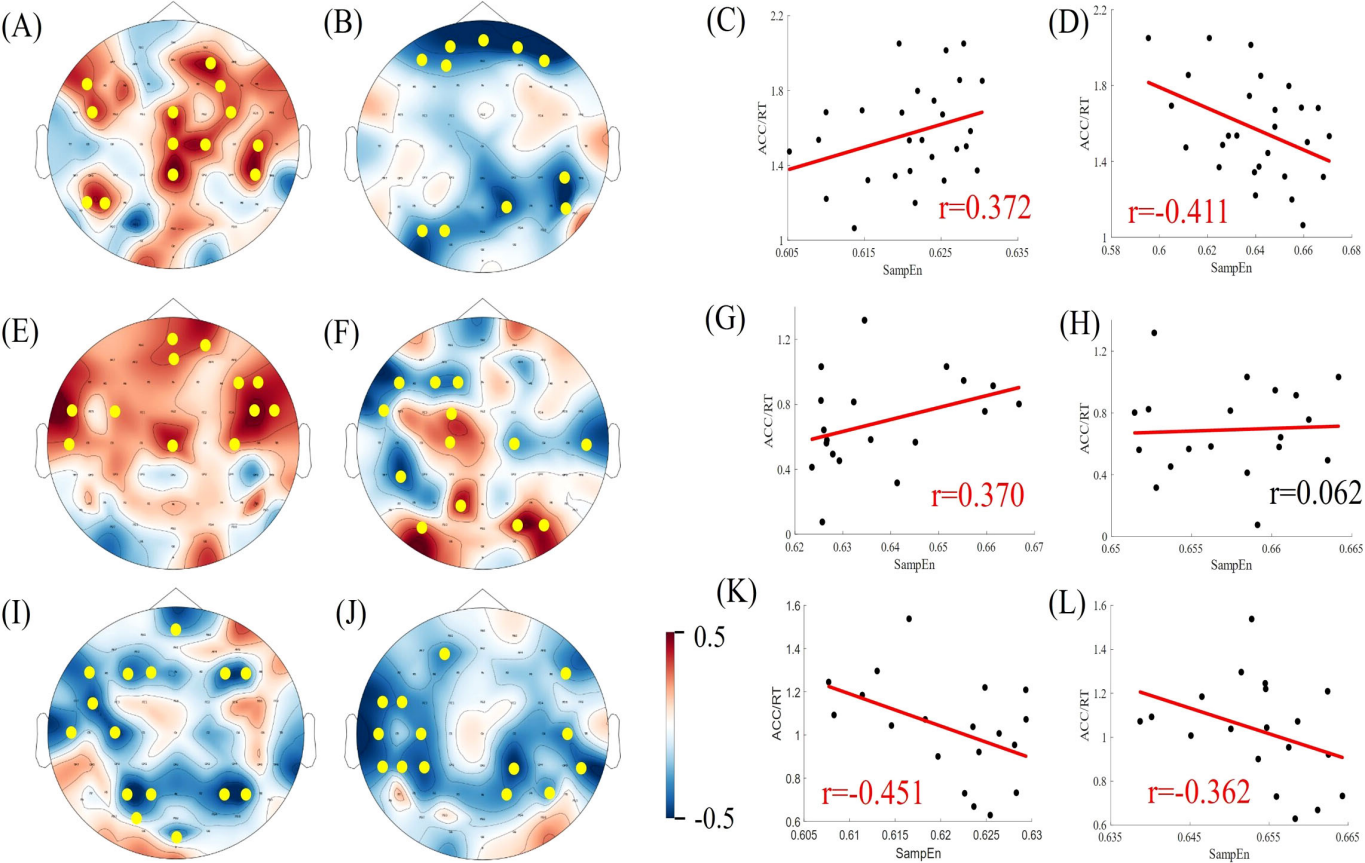
Correlation between EEG complexity during task states and cognitive performance in simple task difficulty. **(A, B, E, F, I, J)** Topographic maps of Spearman correlation coefficients between alpha/beta band sample entropy and task performance among participants in the healthy, deficit, and non-deficit groups, with yellow dots indicating significant correlations at electrode points. **(C, D, G, H, K, L)** Scatter plots of alpha/beta band sample entropy versus task performance among participants in the healthy, deficit, and non-deficit groups. Task performance of the healthy group positively correlates significantly with alpha band sample entropy and negatively with beta band sample entropy. Task performance of the deficit group positively correlates significantly with alpha band sample entropy, whereas task performance of the non-deficit group negatively correlates significantly with alpha/beta band sample entropy.

**Figure 8:**
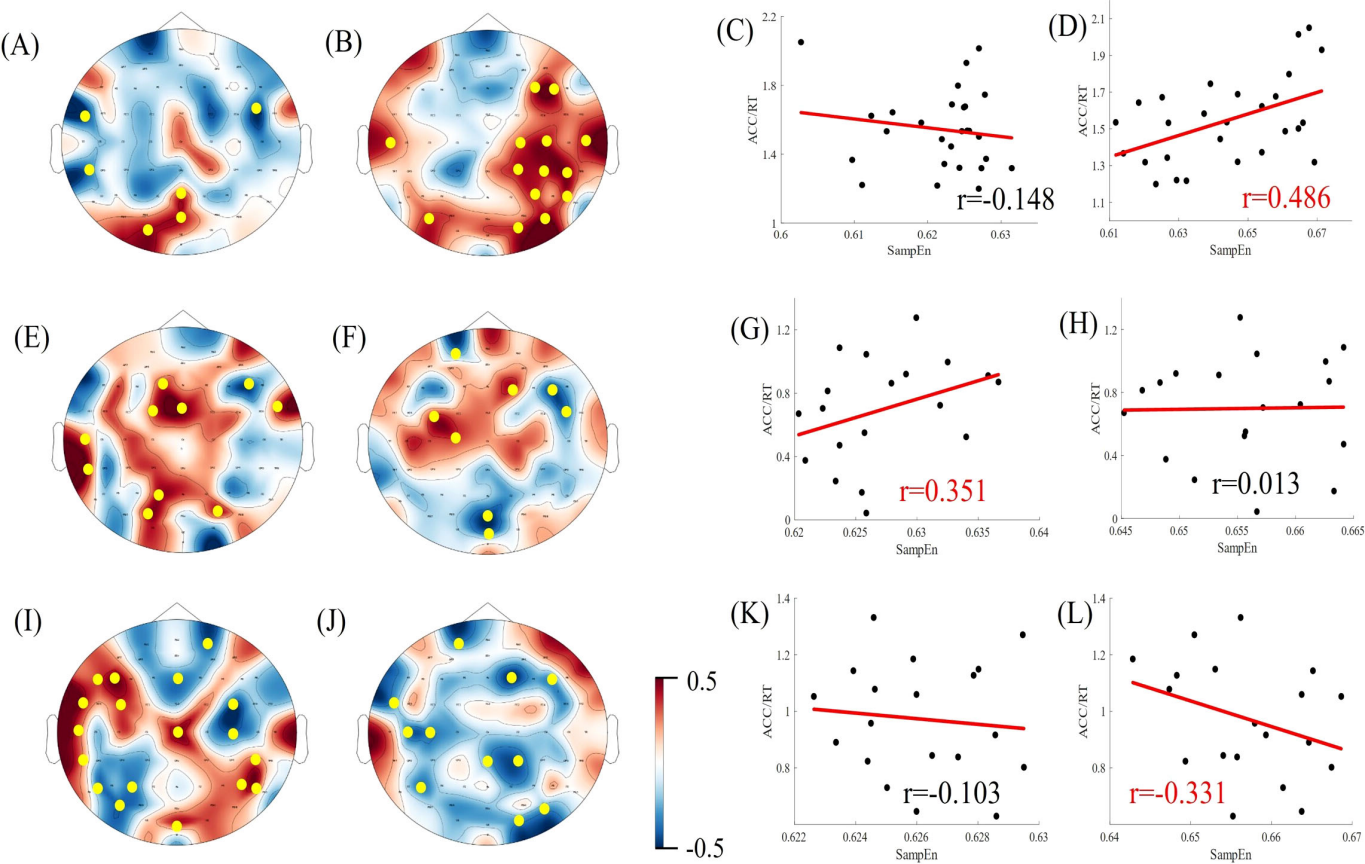
Correlation between EEG complexity during task states and cognitive performance in difficult task difficulty. **(A, B, E, F, I, J)** Topographic maps of Spearman correlation coefficients between alpha/beta band sample entropy and task performance among participants in the healthy, deficit, and non-deficit groups, with yellow dots indicating significant correlations at electrode points. **(C, D, G, H, K, L)** Scatter plots of alpha/beta band sample entropy versus task performance among participants in the healthy, deficit, and non-deficit groups. Task performance of the healthy group positively correlates significantly with beta band sample entropy. Task performance of the deficit group positively correlates significantly with alpha band sample entropy, whereas task performance of the non-deficit group negatively correlates significantly with beta band sample entropy.

Pearson’s correlation coefficients were calculated for subjects' EEG α/β band sample entropy and task performance (ACC/RT) under varying task difficulties within the task state. Task performance was then fitted to the α/β band sample entropy, and the results are presented in **Figures 7**, **8**. Under easy (1T) difficulty conditions, a significant positive correlation was observed between task performance and α-band sample entropy (r = 0.372, *p* < 0.001), while a significant negative correlation was evident between task performance and β-band sample entropy (r = -0.411, *p* < 0.01) in the healthy group. A significant positive correlation was observed between task performance and α-band sample entropy in the deficit group (r = 0.370, *p* < 0.05). The task performance of the non-deficit group was found to be significantly and negatively correlated with alpha/beta band sample entropy (r = -0.451, *p* < 0.01; r = -0.362, *p* < 0.05). Under difficult conditions (4T) of difficulty, task performance in the healthy group was significantly and positively correlated with β-band sample entropy (r = 0.486, *p* < 0.01). The correlation between deficit group task performance and alpha band sample entropy was found to be significant and positive (r = 0.351, *p* < 0.05). Conversely, the correlation between non-deficit group task performance and beta band sample entropy was found to be significant and negative (r = -0.331, *p* < 0.05).

## Discussion

The objective of this study was to undertake a comparative analysis of the multiscale entropy and alpha/beta band sample entropy of the electroencephalogram (EEG) of healthy controls and different subtypes of schizophrenia in both resting and task-state conditions. Furthermore, we examined the relationship between EEG complexity and task performance across different frequency bands. The results demonstrated that there were notable differences in the multiscale entropy of the healthy group, the deficit subtype (DS), and the non-deficit subtype (NDS) in both the resting state and task state conditions. In the task state, the multiscale entropy of parietal regions was significantly lower in the deficit group than in the healthy group, and the multiscale entropy of whole brain regions was significantly higher in the non-deficit group than in the healthy group. A significant difference was observed in the sample entropy of the α-band in occipital regions among the three groups of subjects in the resting state. Furthermore, a significant difference was noted in the sample entropy of the β-band in parietal regions among the deficit and healthy groups. Additionally, a significant difference was identified in the sample entropy of the β-band in whole-brain regions among the non-deficit and healthy groups in the task state. In the healthy group, simple difficulty task performance was positively correlated with α-band sample entropy and negatively correlated with β-band sample entropy, while difficult difficulty task performance was positively correlated with β-band sample entropy. In the DS group, both simple and difficult difficulty tasks Performance was found to be positively correlated with α-band sample entropy. In the NDS group, simple difficulty task performance was negatively correlated with both α-band and β-band sample entropy, while difficult difficulty task performance was negatively correlated with β-band sample entropy. The present study elucidated that deficit and non-deficit schizophrenia patients show different patterns of abnormalities in EEG complexity, suggesting that cognitive impairments in schizophrenia subtypes are caused by different pathogenic mechanisms.

The present study offers an explanation for the inconsistent results observed in previous studies on EEG complexity in schizophrenia. Some studies have reported a reduction in EEG complexity in patients with schizophrenia in comparison to healthy controls (38–40), whereas others have reported an increase in EEG complexity in patients with schizophrenia (6, 41). As presented in the Results section, sample entropy in the beta band was significantly higher in the non-deficit schizophrenia patients than in the healthy group, and sample entropy in the beta band was significantly lower in the deficit schizophrenia patients than in the healthy group at certain electrode points in the frontal region (Cz, FCz, FC1, F4). Previous studies did not differentiate between different subtypes of schizophrenic patients, and also did not explore in detail the existence of significant differences in sample entropy in different brain regions. These facts may account for the contradictory results of the study. Prior research has indicated a correlation between elevated EEG complexity and enhanced information processing (42). Multiscale entropy analyses of different subtypes of the healthy group and schizophrenia demonstrated that discrepancies in EEG complexity were predominantly evident at the middle and high scales, which is concordant with the notion that schizophrenia entails the disconnection of connections between disparate functional networks (16). The SZ patients who exhibited deficiencies demonstrated significantly diminished EEG complexity in comparison to both the healthy and nondeficit patient groups. This finding suggests a reduction in connectivity between their remote neural networks and a concomitant weakening of information processing (43–45).

Previous studies have proposed that EEG complexity is associated with both local functional specialisation and global functional integration (46). However, further investigation is required to elucidate the specific implications of EEG complexity across different frequency bands. In contrast with previous research indicating a correlation between age, cognitive impairment and psychiatric disorders with a reduction in EEG complexity (47, 48), our findings suggest a different pattern. Individuals with primary negative symptoms had lower beta-band complexity in frontal regions compared to healthy controls, which partly supports the hypothesis of frontal-parietal dysfunction in deficit schizophrenia (49). In contrast, the NDS group exhibited divergent patterns compared to the healthy control group, with elevated β band complexity observed across the entire brain during task states. An increase in task performance was associated with a reduction in α and β band complexity. A higher level of complexity is often associated with chaotic and meaningless brain activity (50, 51), which may explain the positive symptoms observed in non-deficit patients (hallucinations and delusions). Moreover, previous research has indicated a correlation between EEG complexity and sustained attention and cognitive flexibility (52), suggesting that the inability of non-deficit patients to maintain attention may be associated with elevated EEG complexity.

High and low frequency temporal oscillations establish precise temporal correlations between distributed neuronal ensembles. High-frequency oscillations involve synchronisation of local brain networks, whereas low-frequency oscillations tend to establish synchronisation over longer distances more often (53, 54). Cognitive performance in the healthy group was found to be positively correlated with central area α-wave complexity and negatively correlated with prefrontal β-wave EEG complexity. This result may indicate an antagonistic relationship between the cognitive functions involved in alpha oscillations and beta oscillations, respectively, when performing working memory tasks. The correlations between cognitive performance and EEG complexity differed between SZ patients with and without deficiencies. In the former group, cognitive performance was positively correlated with alpha-band EEG complexity, while in the latter group, it was negatively correlated with alpha/beta complexity. This may indicate that the deficit and non-deficit types contribute to cognitive impairment via a different pattern of neural network connectivity. Deficit patients appear to have lower connectivity within short-distance neuronal ensembles as well as long-distance neural networks, while non-deficit patients have overly diffuse connections between neural ensembles.

### Limitation

Patients with schizophrenia display a broad spectrum of cognitive impairments, with the change perception task employed in the present study primarily assessing working memory capacity. In future studies, a broader psychological paradigm could be used to identify differences in brain physiological activity between schizophrenics and the general population. In addition, the small sample size may not have been effective in detecting actual effects or differences, increasing the probability of Type II errors, and Bonferroni corrections may have resulted in a loss of significance. Larger sample sizes are needed for further analyses in the future.

## Conclusion

The study revealed discrepancies in EEG complexity between deficit and non-deficit schizophrenic patients and healthy individuals. Additionally, significant variations in EEG complexity were observed across different frequency bands. Furthermore, distinct correlations between task performance and EEG complexity were identified in the three subject groups. The results indicate that the brains of deficit and non-deficit schizophrenics exhibit disparate patterns of physiological aberrations, with cognitive impairments resulting from distinct causative mechanisms. This may facilitate the development of more targeted treatments in the future (55, 56). For example, personalised diagnosis and classification can be achieved through EEG features. Complex EEG can be used as a biomarker to monitor patients' cognitive function, helping clinical doctors understand patients' cognitive status and adjust treatment strategies. And explore the use of drugs or neuromodulation methods to regulate specific frequency bands of brainwave activity, helping to reduce patients' symptoms.

## Data Availability

The raw data supporting the conclusions of this article will be made available by the authors, without undue reservation.
